# Molecular characterisation of infectious bursal disease virus in Namibia, 2017

**DOI:** 10.4102/ojvr.v86i1.1676

**Published:** 2019-07-04

**Authors:** Umberto Molini, Gottlieb Aikukutu, Juliet Kabajani, Siegfried Khaiseb, Giovanni Cattoli, William G. Dundon

**Affiliations:** 1 Department of Pathobiology, School of Veterinary Medicine, Faculty of Agriculture and Natural Resources, University of Namibia, Neudamm Campus, Namibia; 2 Central Veterinary Laboratory, Windhoek, Namibia; 3 Animal Production and Health Laboratory, Joint FAO/IAEA Division of Nuclear Techniques in Food and Agriculture, International Atomic Energy Agency, Siebersdorf, Austria

**Keywords:** poultry, infectious bursal disease virus, Gumboro, Namibia, phylogenetic analysis, VP1, VP2

## Abstract

Between July and September 2017, samples collected from six unvaccinated chickens in Namibia were shown to be positive for infectious bursal disease virus (IBDV) by RT-PCR. Partial sequence and phylogenetic analysis of the VP1 and VP2 genes from six viruses revealed that they all belong to the very virulent pathotype (Genogroup 3) and are genetically very similar to IBDVs identified in neighbouring Zambia. This is the first molecular characterisation of IBDV in Namibia and has implications on the control and management of the disease in the country.

## Introduction

Infectious bursal disease (IBD), also commonly known as Gumboro disease, is a highly contagious immunosuppressive viral disease in poultry. The disease is caused by the IBD virus which is a member of the *Birnaviridae* family of the *Avibirnavirus* genus and infects lymphocytes in the Bursa of Fabricius of the host causing inflammation and subsequent atrophy of the organ. The resulting immunosuppression leaves the birds highly susceptible to secondary infections. The double-stranded RNA genome of IBDV consists of two segments (A and B). Segment A encodes four viral proteins (VP), namely, VP2, VP3, VP4 and VP5, while segment B encodes VP1. Both VP1 and VP2 have been found to contribute to the virulence of IBDV (Boot et al. 2005; Gao et al. 2014; Liu & Vakharia 2004; Yu et al. 2013) and segments of the VP1 and VP2 genes have been used to differentiate IBDVs by phylogenetic analysis (Jackwood 2004).

Two serotypes of IBDV have been identified. Serotype-1 is pathogenic to chickens, while serotype-2 is non-pathogenic (Ismail et al. 1988). Traditionally, serotype-1 viruses have been further classified into classical virulent, very virulent (vv), antigenic variant and attenuated strains. A more recent classification system proposed by Michel and Jackwood (2017) has grouped viruses into seven genotypes based on phylogenetic analysis of the hypervariable region of VP2 (Michel & Jackwood 2017).

Infectious bursal disease is regularly reported in Africa, and circulating viruses have been well characterised in a number of countries, including Egypt, Ethiopia, Nigeria, Tanzania and Tunisia (Jenberie et al. 2014; Kasanga et al. 2007; Mardassi et al. 2004; Nwagbo et al. 2016; Shehata et al. 2017) However, little is known about the genetic makeup of IBDV in southern Africa, with IBDVs having only been genetically characterised in Zambia and South Africa (Kasanga et al. 2013; Ndashe et al. 2016; Vukea et al. 2014).

In the past, incidences of IBD in Namibia have been confined to rural areas. More recently, however, as the poultry industry and chicken populations have grown, the disease has begun to have more impact with significant mortalities being reported throughout the country. As yet, vaccination against IBD is not compulsory in Namibia although live-attenuated D78 virus vaccines are available commercially.

## Material and methods

The samples analysed in this study were collected between July and September 2017 from rural villages in northern Namibia located close to the border with Angola (Table 1). Bursa of Fabricius from dead chickens (3–6 weeks old) that had previously shown signs of illness, including depression, diarrhoea and prostration, were submitted to the Central Veterinary Laboratory, Windhoek. RNA was extracted using the Maxwell^®^ 16 LEV SimplyRNA Tissue Purification Kit (Promega) according to the manufacturer’s instructions with an elution volume of 50 *µ*l. A fragment of the VP2 gene was amplified using the One-Step Reverse Transcriptase – Polymerase Chain Reaction Kit (Qiagen). The primer pair IBDV1 (5’ TCAGGATTTGGGATCAGC 3’) and IBDV2 (5’ TCACCGTCCTCAGCTTAC 3’), which produces an amplicon of 640 bp, was used as previously described (Liu, Giambrone & Dormitorio 1994). The following thermal profile was used; reverse transcription at 50 °C for 30 minutes, initial denaturation at 95 °C for 15 min and then 40 cycles of denaturation at 95 °C for 30 seconds, annealing at 55 °C for 45 s and elongation at 72 °C for 45 s, followed by a final elongation at 72 °C for 5 min. For the VP1 gene, primer pair B-Univ-F (5’ AAT GAG GAG TAT GAG ACC GA 3’) and B-Univ-R (5’ CCT TCT CTA GGT CAA TTG AGT ACC 3’) was used to produce a 1050 bp amplicon (Islam et al. 2012). The amplification conditions were as follows: reverse transcription at 50 °C for 30 min, initial denaturation at 95 °C for 15 min and then 35 cycles of denaturation at 95 °C for 10 s, annealing at 58 °C for 90 s and elongation at 68 °C for 30 s, followed by a final elongation at 68 °C for 7 min. Amplified fragments were visualised on 1.0% – 1.5% agarose gels. Positive RT-PCR amplicons were purified using a QIAquick PCR purification Kit (Qiagen) and were sent to LGC Genomics (Berlin, Germany) for sequencing. All sequences generated were deposited in GenBank under accession numbers MH237850 to MH237861. The Staden Package (http://staden.sourceforge.net/) was used to assemble the generated sequences. Multiple sequence alignment was performed using MUSCLE (http://www.ebi.ac.uk/Tools/msa/muscle/) with default settings, incorporating all the sequences generated here combined with a selection of representative sequences available in GenBank. Phylogenetic trees were estimated using the neighbour-joining method available in MEGA 6 (Tamura et al. 2013), employing the maximum composite likelihood model of nucleotide substitution and 1000 bootstrap replications.

**TABLE 1:** Description of samples analysed in this study.

Sample	Location	Date	Vaccination status	Birdtype	Age (week)	Breed	Flock size (*n*)	Lesions	Mortality (%)
G1	Ohangwena	14-09-2017	Unvaccinated	Broiler	3	Rhode Island Red	36	Swollen and oedematous bursa	30
G2	Ohangwena	14-09-2017	Unvaccinated	Broiler	3	Rhode Island Red	19	Swollen and oedematous bursa	25
G5	Kunene	21-07-2017	Unvaccinated	Broiler	6	Sussex	42	Haemorrhagic bursa	90
G10	Oshikoto	24-08-2017	Unvaccinated	Broiler	4	Sussex	21	Swollen and oedematous bursa	50
G12	Omusati	14-09-2017	Unvaccinated	Broiler	6	Rhode Island Red	27	Haemorrhagic bursa	90
5707	Oshana	04-09-2017	Unvaccinated	Broiler	5	Rhode Island Red	33	Swollen and oedematous bursa	70

### Ethical considerations

This article followed all ethical standards for a research without direct contact with human or animal subjects.

## Results and discussion

**FIGURE 1:**
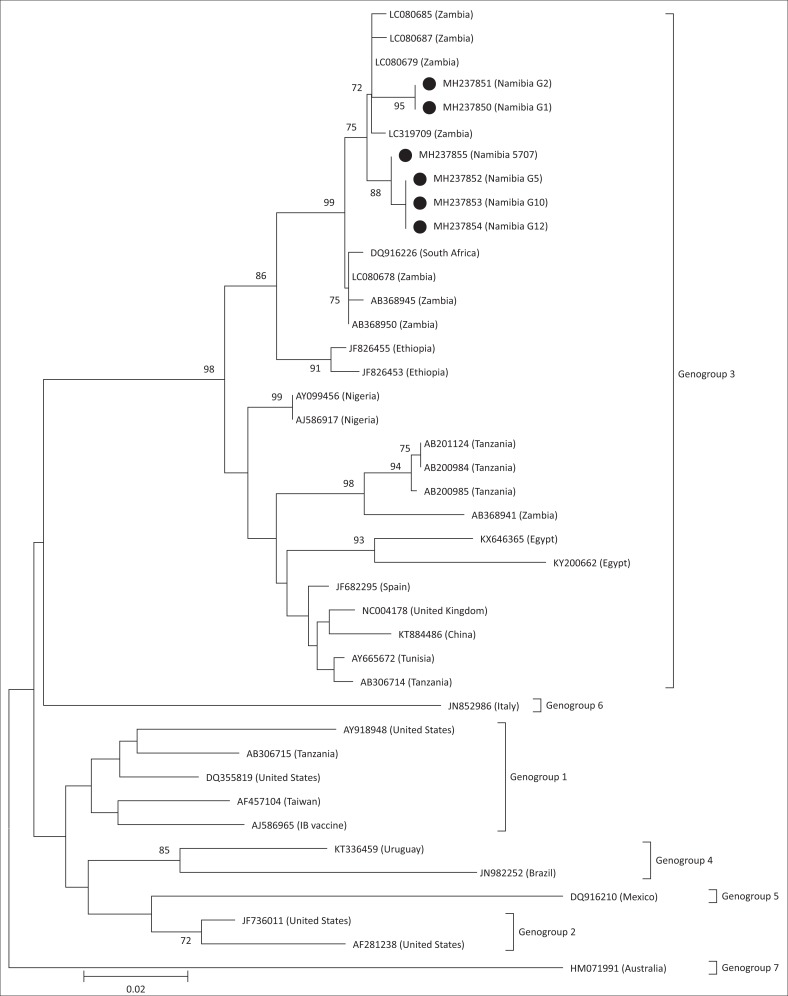
Neighbour-joining analysis using the MEGA6 software of a partial nucleotide sequence (403 bp) of the VP2 gene from the infectious bursal disease virus samples investigated (filled circles) and representative sequences from GenBank. The numbers indicate the bootstrap values calculated from 1000 bootstrap replicates. The scale bar represents nucleotide substitutions per site. The different Genogroups are indicated as described by Michel and Jackwood (2017).

From the phylogenetic analysis of the VP2, it can be seen that the viruses from Namibia clustered in Genogroup 3 (vvIBDV) along with other viruses identified in Africa, Asia and Europe (Michel & Jackwood 2017) (Figure 1). It was also evident from the phylogenetic analysis that the viruses were genetically similar to viruses identified in Zambia and South Africa as opposed to those identified in other African countries (e.g. Ethiopia, Nigeria, Egypt, Tunisia and Tanzania). Interestingly, there was some genetic variation between the Namibian viruses; samples G1 and G2 were identical and clustered together but were genetically distinct, with a 1.3% – 1.5% nucleotide divergence, from samples G5, G10, G12 and 5707. This implies that G1 and G2 do not share a recent common origin with samples G5, G10, G12 and 5707. Samples G1 and G2 were collected in the Ohangwena region of Namibia which borders Angola. There are several authorised live bird markets in this border region in which poultry originating from Angola are both bought and sold. These markets may have been a source of viruses G1 and G2. However, Namibia, also shares a border with Zambia in the north-east of the country. Given the similarity of the Namibian samples with IBDV from Zambia, as shown by the phylogenetic analysis, the two countries may also share a common origin or source of the virus. Nonetheless, given that there is no published genetic data on IBDV available from Angola, or for that matter from Botswana or Zimbabwe, despite disease occurrence being reported (Kelly et al. 1994; Mushi et al. 2006), no conclusion can yet be made on the origin of the IBDV investigated in this study.

Owing to the segmented nature of the IBDV genome, genome reassortment and recombination events, that have been shown to increase the virulence of the virus in addition to altering viral antigenicity, have been reported (Boot et al. 2005; Brandt et al. 2001; Gao et al. 2014; Jackwood & Sommer-Wagner 2011; Liu & Vakharia 2004; Wei et al. 2008; Yu et al. 2013). Kasanga et al. (2013) identified a natural reassortant IBDV (KZC-104) in Zambia in 2004 that consisted of a very virulent segment A and a classical attenuated segment B. The genome segments of the more recently identified viruses from Zambia by Ndashe et al. (2016) were both very virulent.

**FIGURE 2:**
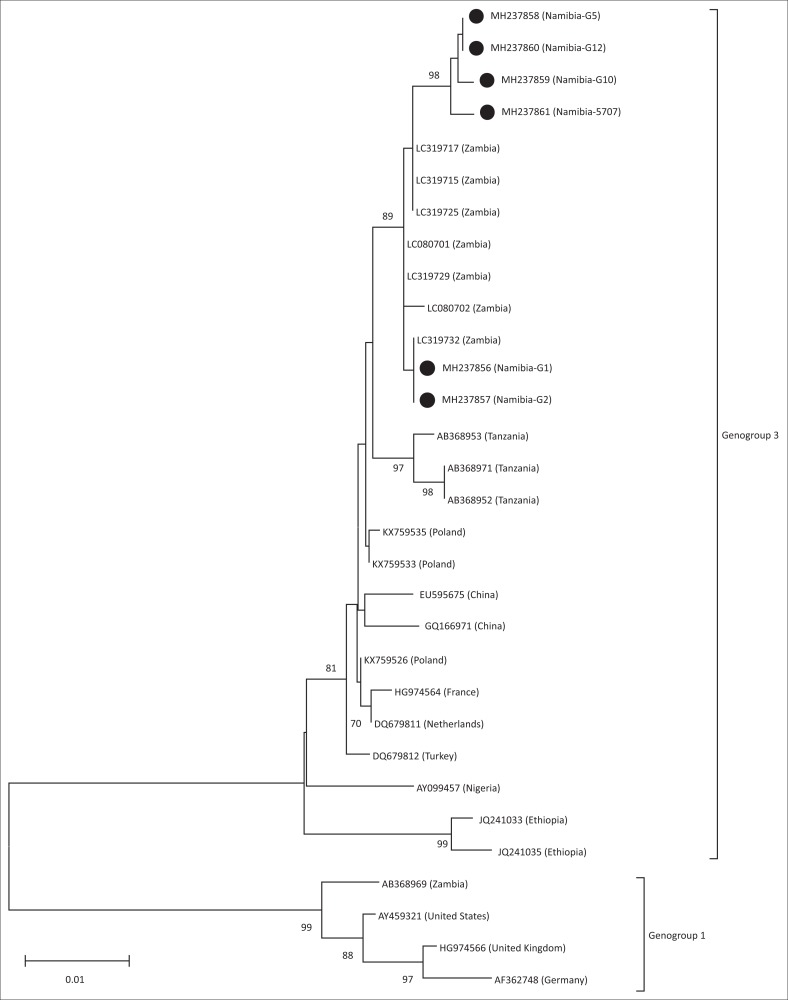
Neighbour-joining analysis using the MEGA6 software of a partial nucleotide sequence (688 bp) of the VP1 gene from the infectious bursal disease virus samples investigated (filled circles) and representative sequences from GenBank. The numbers indicate the bootstrap values calculated from 1000 bootstrap replicates. The scale bar represents nucleotide substitutions per site. The different Genogroups are indicated as described by Michel and Jackwood (2017).

Therefore, to determine whether the Namibian IBDVs were reassortants or not, a segment of the VP1 gene from the six samples was amplified by RT-PCR and sequenced. The results of the phylogenetic analysis of the VP1 segment shown in Figure 2 clearly showed that the Namibian viruses were not reassortants and were more similar to the Genogroup 3 viruses described by Ndashe et al. (2016) and not the Genogroup 1 KZC-104 (GenBank AB368969) reassortant described by Kasanga et al. (2013). The tree also confirmed that samples G1 and G2 were genetically divergent (1.0% – 1.2 %) from samples G5, G10, G12 and 5707.

The predicted amino acid sequence of the partial VP2 and VP1 was checked for characteristic aa residues. For VP2, all of the viruses possessed typical aa residues seen in other vvIBDV (Genogroup 3), namely, 222A, 242I, 253Q, 256I, 294I and 299S (Brandt et al. 2001; Ndashe et al. 2016) (Table 2).

**TABLE 2:** Comparison of amino acid residues of the VP2 between the samples investigated and other infectious bursal disease viruses.

Genogroup	Virus	Amino acid position						
		222	242	253	256	294	299	300
3	Namibia G1	A	I	Q	I	I	S	Q
3	Namibia G2	A	I	Q	I	I	S	Q
3	Namibia G5	A	I	Q	I	I	S	Q
3	Namibia G12	A	I	Q	I	I	S	Q
3	Namibia G10	A	I	Q	I	I	S	Q
3	Namibia 5707	A	I	Q	I	I	S	Q
3	LC080685 (Zambia)	A	I	Q	I	I	S	Q
3	LC080687 (Zambia)	A	I	Q	I	I	S	Q
3	LC080679 (Zambia)	A	I	Q	I	I	S	Q
3	LC319709 (Zambia)	A	I	Q	I	I	S	Q
3	DQ916226 (South Africa)	A	I	Q	I	I	S	Q
3	LC080678 (Zambia)	A	I	Q	I	I	S	Q
3	AB368945 (Zambia)	A	I	Q	I	I	S	Q
3	AB368950 (Zambia)	A	I	Q	I	I	S	Q
3	JF826455 (Ethiopia)	A	I	Q	I	I	S	Q
3	JF826453 (Ethiopia)	A	I	Q	I	I	S	Q
3	AY099456 (Nigeria)	A	I	Q	I	I	S	E
3	AY586917 (Nigeria)	A	I	Q	I	I	S	E
3	AB201124 (Tanzania)	A	I	Q	I	I	S	A
3	AB200984 (Tanzania)	A	I	Q	I	I	S	A
3	AB200985 (Tanzania)	A	I	Q	I	I	S	A
3	AB368941 (Zambia)	A	I	Q	I	I	S	A
3	KX646365 (Egypt)	A	I	Q	I	I	S	E
3	KY200662 (Egypt)	A	I	Q	I	I	S	E
3	JF682295 (Spain)	A	I	Q	I	I	S	E
3	NC004178 (United Kingdom)	A	I	Q	I	I	S	E
3	KT884486 (China)	A	I	Q	I	I	S	E
3	AY665672 (Tunisia)	A	I	Q	I	I	S	E
3	AB306714 (Tanzania)	A	I	Q	I	I	S	E
6	JN852986 (Italy)	Q	V	E	K	L	S	E
1	AY918948 (United States)	S	I	Q	A	L	N	E
1	AB306715 (Tanzania)	P	V	Q	I	L	N	E
1	DQ355819 (United States)	P	V	Q	I	I	N	E
1	AF457104 (Taiwan)	S	V	Q	I	L	N	E
1	AJ586965 (IB Vaccine)	P	V	H	I	L	N	E
4	KT336459 (Uruguay)	S	V	Q	V	L	S	E
4	JN982252 (Brazil)	S	V	Q	V	L	N	E
5	DQ916210 (Mexico)	T	V	Q	V	L	N	E
2	JF736011 (United States)	T	V	Q	V	L	N	E
2	AF281238 (United States)	T	V	Q	V	L	N	E
7	HM071991 (Australia)	P	V	Q	V	L	S	E

A, alanine; I, isoleucine; Q, glutamine; S, serine; V, Valine; E Glutamic acid; T, threonine; P, proline; N, asparagine; K, lysine; L, leucine.

Likewise, the VP1 aa sequences possessed residues 145T, 146D, 147N, 242E and 287A. The TDN triplet has been shown by Gao et al. (2014) to contribute to viral virulence, while 242E and 287A have been identified as possible virulent determinants (Yu et al. 2010). At amino acid position 119 of the VP1, samples G5, G12 and 5707 possessed an Aspartic acid (D) residue, while samples G1, G2, G10 and all of the other viruses included in the phylogenetic analysis possessed a Glutamic acid (E) residue at this position. In addition, at amino acid position 252 of VP1, samples G5, G10, G12 and 5707 differed from all of the other viruses (including G1 and G2) by possessing Valine (V) instead of an Isoleucine (I) (Table 3). The significance of these amino acid differences is unknown at present and requires further investigation.

**TABLE 3:** Comparison of amino acid residues of the VP1 between the samples investigated and other infectious bursal disease viruses.

Genogroup	Virus	Amino acid position						
		119	145	146	147	242	252	287
3	Namibia G1	E	T	D	N	E	I	A
3	Namibia G2	E	T	D	N	E	I	A
3	Namibia G5	D	T	D	N	E	V	A
3	Namibia G12	D	T	D	N	E	V	A
3	Namibia G10	E	T	D	N	E	V	A
3	Namibia 5707	D	T	D	N	E	V	A
3	LC319717 (Zambia)	E	T	D	N	E	I	A
3	LC319715 (Zambia)	E	T	D	N	E	I	A
3	LC319725 (Zambia)	E	T	D	N	E	I	A
3	LC080701 (Zambia)	E	T	D	N	E	I	A
3	LC319729 (Zambia)	E	T	D	N	E	I	A
3	LC080702 (Zambia)	E	T	D	N	E	I	A
3	LC319732 (Zambia)	E	T	D	N	E	I	A
3	AB368953 (Tanzania)	E	E	D	N	E	I	A
3	AB368971 (Tanzania)	E	T	D	N	E	I	A
3	KX759535 (Poland)	E	T	D	N	E	I	A
3	KX759533 (Poland)	E	T	D	N	E	I	A
3	EU595675 (China)	E	T	D	N	E	I	A
3	GQ166971 (China)	E	T	D	N	E	I	A
3	KX759526 (Poland)	E	T	D	N	E	I	A
3	HG974564 (France)	E	T	D	N	E	I	A
3	DQ679811 (Netherlands)	E	T	D	N	E	I	A
3	DQ679812 (Turkey)	E	T	D	N	E	I	A
3	AY099457 (Nigeria)	E	T	D	N	E	I	A
3	JQ241033 (Ethiopia)	E	T	D	N	E	I	A
3	JQ241035 (Ethiopia)	E	T	S	N	E	I	A
1	AB368969 (Zambia)	E	N	E	G	D	I	T
1	AY459321 (United States)	E	N	E	D	D	I	T
1	HG974566 (United Kingdom)	E	N	E	G	D	I	T
1	AF362748 (Germany)	E	N	E	G	D	I	T

E, glutamic acid; T, threonine; D, Aspartic acid; S, serine; N, asparagine; I, isoleucine; A, alanine; G, glycine; V, Valine.

## Conclusion

This study has identified, for the first time in Namibia, IBDVs that are genetically similar to viruses identified in neighbouring Zambia. Combined with data from further molecular epidemiological investigations throughout Namibia and the region (including Angola, Botswana and Zimbabwe), this study will allow for the design of targeted vaccination programmes and strategies by Namibian veterinary authorities. It has also generated comparative data for those interested in the circulation of IBDV in southern Africa.
